# A Study Evaluating the Accuracy of Triage for Breast Referrals During the Covid-19 Pandemic in a Tertiary Hospital

**DOI:** 10.1089/whr.2023.0021

**Published:** 2023-08-16

**Authors:** Ngee-Ming Goh, Claudiu Simonca, Maria Verroiotou, Stephane Jenkins

**Affiliations:** ^1^ Primrose Breast Unit, University Hospitals Plymouth NHS Trust, Derriford, Plymouth, United Kingdom.; ^2^ The Breast Care Unit, Medway Maritime Hospital, Gillingham, United Kingdom.

**Keywords:** COVID, triage, breast referral

## Abstract

**Objective:**

To evaluate the accuracy of breast referral triage during Covid-19.

**Design:**

Retrospective case study.

**Setting:**

Primrose Breast Unit, Derriford Hospital, Plymouth. From March 17th to June 30th to encompass the height of the pandemic and the early enforced changes to practice.

**Participants:**

All referrals received, triaged, and seen (*n* = 870) in the unit, identified by referral records.

**Main Outcome Measures:**

The primary outcome measure of a positive disease state was of a histological diagnosis of cancer, with the absence of a cancer diagnosis representing a negative disease state. Accuracy has been determined by sensitivity and specificity calculations; thus defined by correctly triaging cancers to face-to-face clinics and benign cases to telephone or video clinics.

**Results:**

Sixty-eight cancers (7.8% of referrals) were detected after initial triage and consultation, of which 51 (sensitivity = 75%) were triaged to one-stop-clinic; positive predictive value was 18.89%. Eight hundred two (specificity = 72.69%) of benign cases were triaged to phone or video clinic initially; negative predictive value was 97.15%. Comparing the study's incidence of cancer (7.8%) to the preceding year's (2019) of 6.8% with Yate's correction shows no significant difference (*p* < 0.05).

**Conclusion:**

Triage accuracy is sufficiently robust to diagnose cancer promptly, which should reassure clinicians and decision makers within the cancer networks.

## Background

Breast cancer is the most common cancer in the UK, with increasing incidence rates since the 1990s and now over 55,000 new cases a year. This corresponds to over 11,000 breast cancer deaths every year. Breast cancer is the fourth most common cause of cancer death in the UK and the second most common among women.^1^ Unsurprisingly it has therefore been at the forefront of the NHS Cancer Plan to introduce standards regarding waiting times. This resulted in a “2 week wait” (2ww) standard pathway for urgent General Practitioner (GP) referrals for suspected cancer, as well as a routine referral route which culminated in the “Choose and Book” (C&B) route.^2^

The Cancer Reform Strategy of 2007 noted that despite these standards, treatment varied greatly among breast cancer patients. A major cause for this was that only half of diagnosed breast cancers actually came through the 2ww route, with the other half coming from other referrals or via screening. Only the 2ww patients would actually benefit from the streamlined pathways of care. The strategy thus called for all patients with breast symptoms to be seen within 2 weeks—this standard was formally started on January 1^st^, 2010. The only exclusions were referrals from family history clinics (unless symptomatic) and referrals for cosmetic reasons.^2,3^ This standard has therefore been the target for all breast units until the start of the Covid-19 pandemic.

The Covid-19 pandemic naturally led to the UK health service protecting more vulnerable patients who may be “shielding,” as well as reducing unnecessary footfall in hospitals. The Association of Breast Surgery (ABS) conceded that to continue a normal service in the wake of the pandemic worsening was unrealistic and thus issued a statement offering advice, and covering theater protocol, follow-up, and multidisciplinary team meetings, advice on referrals and triage was also included.^4^

The statement also advised that patients should self-isolate for 7 days before their clinic appointment, to which they could only bring one accompanying person. If they had potential Covid symptoms, they were to phone and rebook their appointment. Breast units were advised to triage all referrals, with the following recommendations:

- “See only referrals where there is a higher index of suspicion of cancer, providing there are staff to run clinics.
- Write to or phone referrals with a lower index of suspicion of cancer for example, breast pain.
- Very frail patients, especially if in nursing homes, referred with suspicious lumps should not be seen in clinic until the situation has changed. If the Government introduces self-isolation for people aged 70 years and older, then consideration should be given as to whether these patients should be seen in the clinic. Older patients especially with comorbidities are at highest risk of death from coronavirus and they should be seen once the pandemic is over. Start on endocrine therapy empirically.^4^”

A further statement in April 2020 did not substantially change the recommendations. However, the May 2020 statement suggested that the lower risk referrals, such as breast pain or bilateral nipple discharge in patients younger than 30 years of age, could be triaged by telephone and potentially discharged back to their GP or deferred imaging arranged.^5^ A further recommendation in October 2020 discussed delivering breast services in a “new normal” environment, expanding the low-risk group to include gynecomastia. Furthermore, it reviewed lessons learnt during the pandemic, including that these low-risk patients did not need to have face-to-face consultations and that directing patients and GPs to appropriate and good-quality information could supplement practice and lessen the burden to one-stop clinic.^6^

Given this change in direction to how breast referrals have been managed, it is therefore pertinent to assess its effectiveness. At our institution, patients were triaged by consultant breast surgeons to clinic or phone/video consultation on the basis of the referral letter. Patients with breast pain or cyclical symptoms, especially if young, or older and comorbid patients who were shielding and isolating, were telephoned initially and only brought to clinic if suspicion was raised, or at a later date following the commencement of endocrine treatment. The aim of this study is to assess the sensitivity and specificity of this process as a method of validating this system change.

## Methods

Data were collected from electronic records retrospectively from March 17th to June 30th, 2020. Triage decisions were made in line with the agreed pathway on the South and West Devon Formulary and Referral system, setup by the NHS New Devon Care Commissioning Group.^7^ Suspected cancer referrals were therefore prioritized for face-to-face clinics with the proviso that elderly, frail, or comorbid patients may need to be treated empirically and only physically reviewed once the situation stabilized. Non-urgent referrals would ordinarily be directed toward a phone or video consultation (Table 1).

**Table 1.** Local Referral Criteria

Referral criteria
Suspected cancer referral
Aged 30 years and older with an unexplained breast lump with or without pain
Aged 50 years and older with any of the following symptoms in one nipple only
Discharge (spontaneous clear or bloody)
Retraction (new onset and distortion ???)
Skin changes of concern (e.g. males older than 50 years with unilateral firm subareolar mass with or without nipple distortion or skin changes
Consider suspected cancer referral
Aged older than 30 years with unexplained lump in the axilla and have skin changes that suggest breast cancer
A nonurgent referral should be considered in
Aged younger than 30 years with an unexplained breast lump with or without pain
Women aged younger than 30 years with a lump
Patients with breast pain and no palpable abnormality, when initial treatment fails and/or with unexplained persistent symptoms. (Use of mammography in these patients is not recommended)

**FIG. 1.**
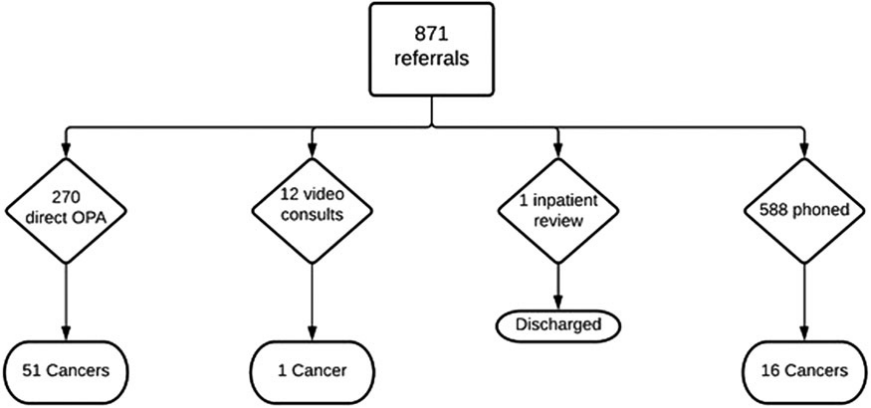
Flowchart depicting all referrals and their subsequent triage route. OPA, outpatient appointment.

All symptomatic breast patient referrals from general practice or hospital inpatient teams, having been triaged to one-stop clinic, phone consultation, or video consultation at Derriford Hospital, United Kingdom, were analyzed. Records were also examined for clinic outcomes and histopathology results for a follow-up period ending August 30, 2021. Patients and the public were not involved in the production of this article (Fig. 1).

## Results

Eight hundred seventy-one referrals were received during this time period and subsequently triaged. Five hundred eighty-eight (67.5%) of referrals were triaged to phone consultation; 270 (31%) were triaged to one-stop clinic; 12 (1.4%) were triaged to video consultation; and 1 (0.1%) was reviewed as an inpatient and discharged – this was excluded having received no triage. In total, 68 (7.8%) cancers were confirmed on histopathology. For the purposes of this study, cancer represents a positive disease state, with the absence of a cancer diagnosis in the follow-up period serving as a negative disease state.

Sensitivity (true positive rate) therefore represents the proportion of cancers correctly triaged to a one-stop clinic. Specificity (true negative rate) represents the proportion of patients without cancer correctly triaged to a phone or video consultation. Positive predictive value (precision) indicates the probability of cancer among those triaged to a one-stop clinic. Negative predictive value (NPV) indicates the probability of a benign/noncancer diagnosis for those triaged to phone or video consultation (Table 2).

**Table 2.** Monthly Breakdown of Referrals and Outcomes

	March	April	May	June	Total
Referrals	88	199	252	331	870
Benign	80	175	239	308	802
Cancer	8	24	13	23	68
Sensitivity	75%	70.83%	76.90%	78.26%	75.00%
Specificity	55%	83.43%	68.20%	74.68%	72.69%
PPV	14.30%	36.96%	11.60%	18.75%	18.89%
NPV	95.70%	96.42%	98.20%	97.87%	97.15%

NPV, negative predictive value; PPV, positive predictive value.

Four patients represented within a year with cancers after being discharged. These patients were all initially triaged to phone clinic, although two were subsequently brought into clinic within 2 weeks. One of those was initially referred with potential fat necrosis. When this had not resolved at a 1-month interval phone consult, a clinic review in 2 weeks was arranged, which with reassuring clinical examination led to discharge. Screening mammography 10 months later led to a stage 3 breast cancer diagnosis. The other had presented with musculoskeletal pain which had resolved by the time of the clinic 2 weeks later. A subsequent medical admission 2 months later for breathlessness detected recurrent, metastatic (axillary, lung, chest) disease without any palpable component.

One patient initially discharged from phone clinic after a breast pain history was eventually re-referred when a mass became palpable after 2 months and was subsequently diagnosed with a breast cancer. Of note, this initial discharge was in line with the “Urgent cancer diagnostic services during Covid-19” document published by NHS England in January 2019, wherein digital information and education can be given if cancer not considered likely following a breast pain referral.^8^ The other patient had been discharged after chest wall blistering and neuropathic pain following a bout of shingles had resolved, only to have a contralateral cancer diagnosis following screening mammography 7 months later. This case in due course required ultrasound wire-localization for the nonpalpable cancer.

In addition, two further cancer diagnoses have been made in patients following triage and discharge from phone clinic, but more than 1 year following initial review. Both cases had reported lumpiness or pain, which had resolved by the time of their phone consultation.

Over the preceding time period from January 02, 2019 to December 31, 2019, of the 5409 symptomatic referrals received, 369 cancers were diagnosed, giving an incidence rate of 6.8%. Contingency table evaluation with Yate's correction comparing this to the incidence of 68 cases from 870 referrals (7.8%) during our study shows that for *p* < 0.05, there is no significant difference.

## Discussion

The referrals included all sources, ranging from GP 2ww and C&B referrals, to inpatient referrals from other teams that were not clinical emergencies.

This study shows that our triage process is sensitive with a high NPV demonstrating its accuracy alongside a slight improvement over time. Seventy-five percent of cancer cases were correctly triaged to a face-to-face clinic, while 25% were initially triaged to a phone or video consultation. For comparison, before the Cancer Reform Strategy of 2007 with doubts rising about the 2ww/routine referral pathways,^9–14^ some studies showed as many as 36% of breast cancer cases not being referred as “urgent.”^15^

In particular, one large study at a neighboring region's tertiary center charted several years of decreasing referral accuracy, with a nadir reached in 2005. At that stage 73% of cancer cases were referred by the 2ww protocol, representing 7.7% of total 2ww referrals. The 27% of cancers detected via the routine pathway in turn represented 5.3% of total referrals.^16^ In contrast, our cancer cases equated to 18.9% of clinic cases and 2.8% of telephone/video consultations.

Despite this, only one case effectively had a delayed diagnosis, proving that the phone/video consultations were sufficiently robust enough to identify potential cancer cases. An overall NPV of 97.15% in turn illustrates how few cancers were coming through the phone/video consultation route.

Interestingly, specificity did vary from month to month, likely as a result of changing attitudes during the pandemic. Specificity peaked in April, before dropping in May and increasing slightly in June as patients became less anxious about attending hospital. In turn, better protocols and systems within the hospital were introduced to allow more direct patient consultations, especially for those previously deemed too vulnerable and/or requiring to shield.

Most importantly, given the limited follow-up period of this study and hence the potential of missed or interval cancers, evaluation has shown no significant difference when compared to our unit's practice pre-Covid. This non-inferiority combined with the demonstrated accuracy should serve to reassure us of the safety of our new practice.

## Conclusion

Overall, these figures can reassure us that cancer diagnosis has been maintained through this new process. Given that some of these measures are likely to remain for the foreseeable future throughout the NHS, it was imperative that this was the case. In turn benefits, such as less demand on face-to-face clinics, resulting in less demand on diagnostic services, are indirect advantages from the novel methods of managing low-risk referrals.

Given the year-on-year^16^ increasing demand on breast services, along with the financial and time costs of one-stop clinics, lessons learnt during this pandemic can be used to optimize our use of scarce resources. While the 2 week wait standard is here to stay, the ideal of a one-stop clinic for all referrals is now questionable. Instead, services can be allocated appropriately by triaging to a variety of clinics with different requirements on surgical, radiology, nursing, and administrative capacity.

Consideration can also be made as to whether the referral/triage model itself can be streamlined for efficiency. Further studies evaluating the gatekeeping/referral role of primary care have been called for^17^ and multivariate predictive models exist that could challenge the manner in which referrals are made and evaluated.^18^

As such, further and ongoing analysis of both the triage process, as well as any new pathways set-up to manage lower risk cases, is vital to ensure that it remains not only vigorous and secure, but also cost-effective and potentially cost saving. Analysis would need to incorporate surveillance of screening records and the national registry to protect against and pick-up interval (missed) cancers. Further studies can also be undertaken to compare our results to those of other breast units around the country.

## Abbreviations Used

ABS: Association of Breast Surgery
C&B: Choose and Book
GP: General Practitioner
NPV: negative predictive value
OPA: outpatient appointment
